# Neutron and Synchrotron Imaging Studies of Preservation State of Metal of Cultural Heritage Objects

**DOI:** 10.3390/jimaging7110224

**Published:** 2021-10-26

**Authors:** Ekaterina Kovalenko, Mikhail Murashev, Konstantin Podurets, Elena Tereschenko, Ekaterina Yatsishina

**Affiliations:** 1 National Research Centre “Kurchatov Institute”, 1 Akad. Kurchatova Sq., 123182 Moscow, Russia; kovalenko_es@mail.ru (E.K.); murashev_mm@nrcki.ru (M.M.); elenatereschenko@yandex.ru (E.T.); yatsishina_eb@nrcki.ru (E.Y.); 2 Federal Scientific Research Center “Crystallography and Photonics”, Russian Academy of Sciences, 119333 Moscow, Russia; 3 Institute for Chemical Reagents and High Purity Chemical Substances, National Research Centre “Kurchatov Institute”, 3 Bogorodskii val St., 123182 Moscow, Russia

**Keywords:** synchrotron tomography, neutron tomography, cultural heritage, preservation, corrosion, funeral pyre

## Abstract

This paper analyzes the results of studies carried out at the National Research Center “Kurchatov Institute”, Moscow, using the methods of neutron and X-ray synchrotron tomography from the point of view of the preservation state of metal objects. Objects damaged by corrosion and exposure to fire were the focus of this study. To identify regions of metal preservation, the diffraction contrast on grains of metal, observed in tomographic projections, was used. The simultaneous use of neutron and synchrotron imaging is shown to be a powerful tool for identification of the constituents of an object.

## 1. Introduction

The investigation of cultural heritage objects using the methods of natural science is important for expanding and deepening their source research capabilities. Such studies enable obtaining new data on culture, science, crafts, technologies, trade relations, and history of entire countries and peoples [1]. One of the important aspects of the study of cultural heritage objects is to obtain information about the physical and chemical impacts to which the material of the object was subjected. Such information can be used to reconstruct the original appearance of an object, to predict its preservation, and to determine the best restoration and conservation strategy. The special value and often fragility of cultural heritage objects require the use of modern nondestructive testing methods for their examination [2,3,4], such as methods based on the propagation of penetrating radiation, i.e., X-rays, synchrotron radiation and neutron beams. With the help of tomography, due to the different attenuation of the radiation beam when passing through materials of different chemical composition, density, and thickness in the object under study, it is possible to obtain three-dimensional information about the internal structure of the object with a spatial resolution at the micron level. At present, the methods of synchrotron and neutron imaging are widely used, carried out on installations of the megascience class. At the National Research Center (NRC) “Kurchatov Institute” in Moscow, Russia, work on the synchrotron and neutron tomography of cultural heritage objects is carried out at the “KSRS-Kurchatov” synchrotron radiation source and the IR-8 research reactor. The purpose of this work is to analyze the results of research previously carried out at the NRC “Kurchatov Institute” from the point of view of studying the preservation state of metal artifacts.

## 2. Methods

Neutron studies were carried out at the IR-8 reactor. Most of the measurements were carried out at the “DRAKON” facility [5] ((NRC) “Kurchatov Institute”, Moscow, Russia), whereas some measurements were conducted on a neutron tomograph [6]. At the DRAKON facility, the neutron beam was formed by a double monochromator with pyrolytic graphite crystals in 002 reflection; the neutron wavelength was 0.175–0.2 nm. Neutron projections were recorded by a position-sensitive detector consisting of a scintillation screen based on a 200 μm thick mixture of ZnS(Ag) and ^6^LiF, a mirror, an objective lens, and a charge-coupled device (CCD) matrix with dimensions of 2048 × 2048. The beam size was 7 × 7 cm. The exposure time was 60–150 s. The spatial resolution of the obtained images was about 200 µm with a pixel size of 65 × 65 µm. When studying objects exceeding the size of the beam, the object was scanned with subsequent stitching of full projections or stitching of tomographic arrays. At the facility [6], the neutron beam was formed by a monochromator with a copper crystal in 111 reflection, the neutron wavelength was 0.152 nm, and a Soller slit was present with a divergence of 20°. Neutron projections were recorded using the same type of position-sensitive detector with a 100 μm thick screen. The beam size was 10 × 10 cm, and the exposure time was 480 s. The spatial resolution was about 160 µm, with a pixel size of 55 × 55 µm.

Synchrotron studies were carried out at the tomographic station on the K6.3 beamline of the Kurchatov synchrotron radiation source “KSRS-Kurchatov”. The spectrum of the synchrotron radiation (SR) from a bending magnet was formed by a copper filter 1.5 mm thick, which gave the SR beam a spectrum maximum of about 56 keV and a spread of about 20 keV. The projections of the object were recorded using a position-sensitive detector consisting of a CsI(Tl) scintillation screen, a mirror, an objective lens, and a 2048 × 2048 CCD matrix. The beam size was 50(H) × 3(V) mm, and the spatial resolution was 75 μm with a pixel size of 25 × 25 μm. The exposure time was 0.5–2 s. Since the SR beam height is small, the projections were compiled from a series of images taken with a shift in height at a step of 1 mm.

ImageJ software package [7] (National Institute of Health, Bethesda, MD, USA) was used for primary image processing, projection assembly, and analysis, whereas Octopus Reconstruction [8] (Inside Matters, Ghent, Belgium) was used for tomographic reconstruction and rendering. Thermal neutron diffraction phase analysis and X-ray fluorescence elemental analysis were used as additional methods.

## 3. Results and Discussion

**Figure 1:**
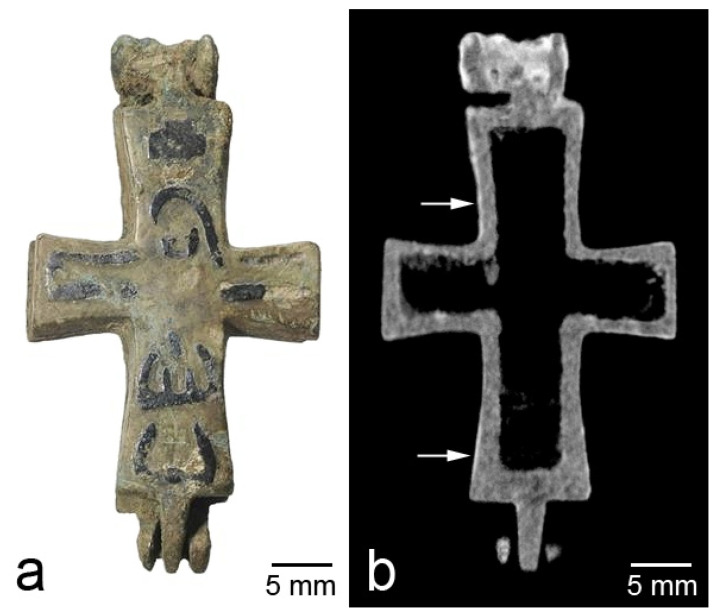
Photograph (**a**) and tomographic slice (**b**) of the cross from Fedosyino-1; arrows show areas of surface corrosion.

Corrosion is the main process that threatens the safety of metal archeological objects. As examples of the detection of corrosion, we refer the reader to tomographic studies of the reliquary cross pendants of the 11th–13th centuries, made by casting from tin–lead bronze [9,10]. These works were mainly aimed at studying the contents of the reliquaries, but the evaluation of the state of the metal was also part of the research task. Various cases were observed; as a rule, it was more or less homogeneous surface corrosion or inhomogeneous corrosion where volumetric areas of damage of the metal took place. An example of the first case is the cross from the rural site of Fedosyino-1 of Suzdal Land (Figure 1). Corrosion products are clearly visible in the tomographic slices as regions of high attenuation due to their content of hydrogen, which has a high thermal neutron attenuation cross-section.

**Figure 2:**
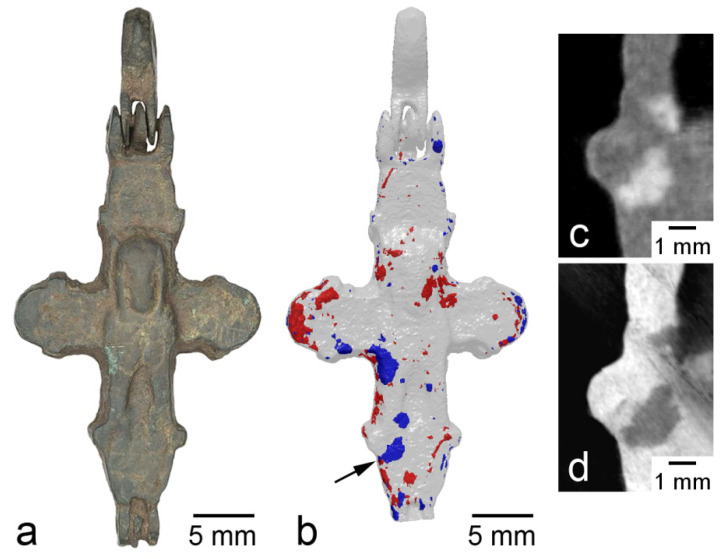
Images of the cross from Soroguzhino-2: (**a**) photograph; (**b**) 3D rendering of the corroded regions, shown in different colors for the different valves; tomography slices of a corrosion region identified in (**b**) by an arrow, taken with neutron (**c**) and synchrotron (**d**) radiation.

**Figure 3:**
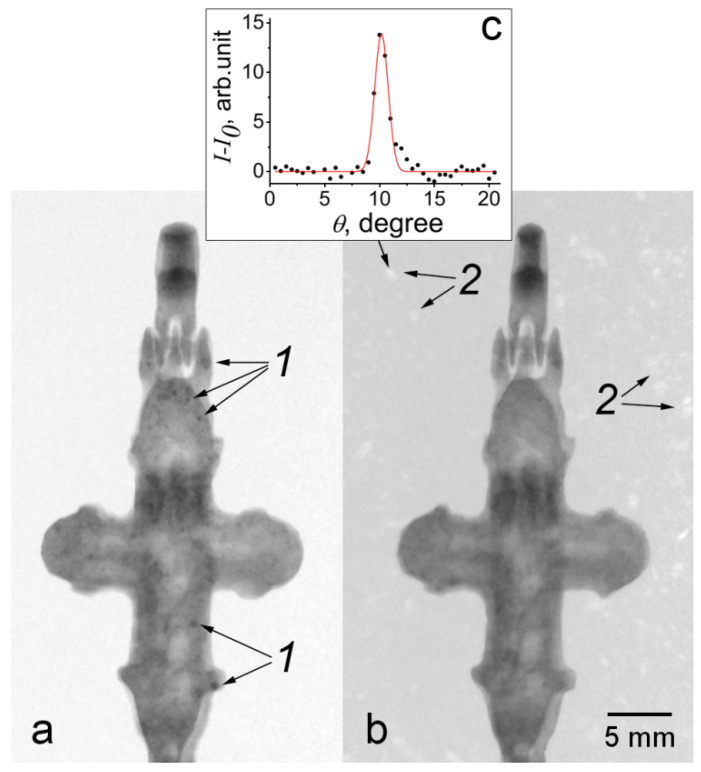
Images of the cross from Soroguzhino-2. Maps of the minimum (**a**) and maximum (**b**) intensity in the angular range of 20° near the frontal view of the cross: 1—images of grains, 2—reflections from grains; a rocking curve of one of the reflections is shown in (**c**).

Inhomogeneous corrosion was observed in the cross from the rural site of Soroguzhino-2, Suzdal Land (Figure 2). It can be seen that the regions of corrosion are 1–5 mm in size and are more or less evenly distributed over the product (Figure 2b). A comparison of neutron and SR images shows that these regions have the opposite contrast; for SR, they are more transparent than metal, due to the fact that the density of the corrosion products is less than the density of the metal (Figure 2c,d). In the stacks of neutron projections of this cross, the diffraction effect of “flickering” is observed. The effect arises due to the strong attenuation of the neutron beam passing through small parts of the volume of the material in a narrow angular range of rotation of the object. This attenuation is due to the fact that, at a certain angular position, a single crystal grain of the metal enters the reflecting position, and a significant part of the beam intensity is removed from the image formation. This diffraction contrast was used, in a more complicated way, for the reconstruction of grain morphology in the polycrysrtalline materials [11]. This effect can also be detected in the following way: considering a sequence of projections in a certain range of angles (up to 20°), and using the ImageJ program function, one can construct a map of the minimum brightness values of these projections. Thus, the regions are distinguished for which the diffraction attenuation of brightness occurs in the given angular range (Figure 3a). These areas are the images of crystallites. The diffractive nature of the contrast in these images is confirmed by the map of maximum values for the same sequence of projections; diffraction reflections are observed in an empty field (Figure 3b). The reflections occur during the sample rotation angle of about 1.5° (full width at half maximum), as shown in Figure 3c. Thus, in the bronze cross with large crystallites of 1–1.5 mm in size, nonuniform internal corrosion is observed, whereas, in the crosses with small crystallites (less than the spatial resolution), surface corrosion is observed. Whether these phenomena are related is not yet clear.

**Figure 4:**
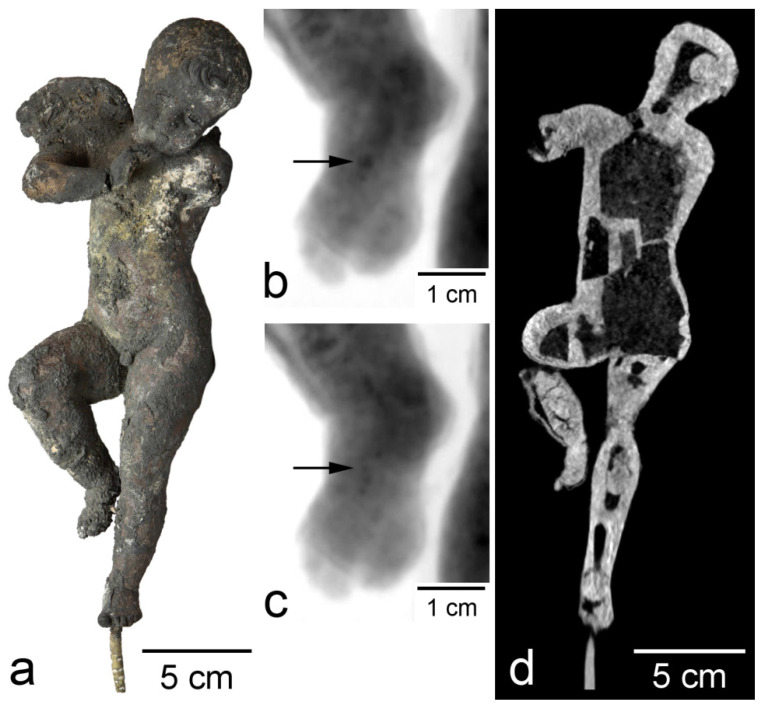
Study of “Dancing Cupid” statuette: (**a**) photograph of the figurine; (**b**,**c**) neutron projections of a fragment of the statuette, obtained with a rotation of 2° (the arrow points to one of the grains); (**d**) tomographic slice.

As an example of the destructive effect of fire, consider the study of the “Dancing Cupid” statuette (Figure 4a) from the collection of the A.S. Pushkin State Museum of Fine Arts, Moscow (Pushkin Museum) [12]. The statuette entered the Pushkin Museum in 1946 among looted art. It was previously stored in Berlin and suffered a fire in May 1945 [13]. Due to significant damage, it was kept in the storerooms of the Moscow museum for a long time, presenting a difficult problem from the point of view of restoration. The “Dancing Cupid” statuette is cast from tin–lead bronze. It has a height of 26.5 cm. The left arm and wing are practically lost, while the right ones are damaged. The color is mainly black, and no traces of patina are observed. In some places, the surface has bulges, and the surface is also covered with spots of various colors. External examination of the figurine raised concerns that the damage affected a significant part of its material. The figurine was examined using neutron tomography. In the neutron projections of the “Dancing Cupid” statuette, the diffraction effect of “flickering” was observed throughout its entire volume (Figure 4b,c). This phenomenon led to the conclusion that almost all the material of the figurine consists of metal, and the fear of oxidation of a significant part of the material was not confirmed. The average grain size was about 500 μm, although individual grains larger than 1 mm were observed. The appearance of large crystallites of copper oxide was not possible due to the higher melting point in comparison with the metal. Neutron tomography of the “Dancing Cupid” statuette shows significant internal damages caused by the fire. Basically, they are expressed in the detachment of a part of the material, and the number of such defects increases in the direction from the head to the feet. The shin of the figurine’s right leg suffered most; cracking occurred not only near the surface, but also in the volume of the metal (Figure 4d).

**Figure 5:**
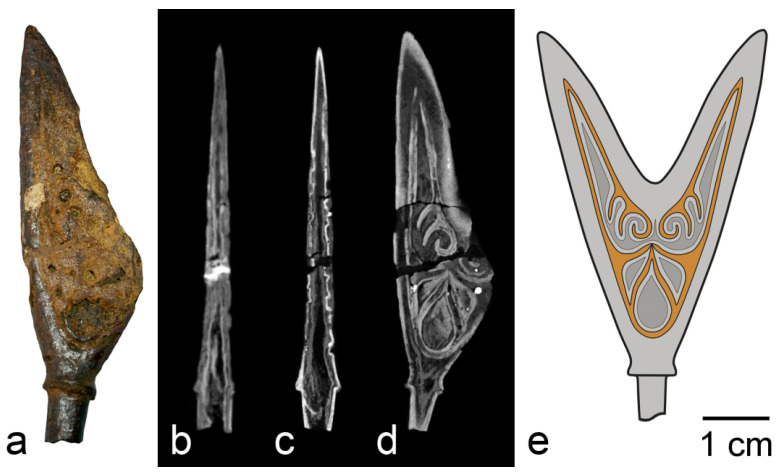
Study of the arrowhead: (**a**) photograph of the object; (**b**) neutron tomographic slice; (**c**) SR tomographic slice; (**d**) SR tomographic slice in plane of ornament; (**e**) reconstruction of initial appearance of the arrowhead.

As an example of the protective effect of fire, the reader can consider the study of an iron arrowhead from the Chernaya Mogila (Black Grave) burial mound [14]. The mound is a 10th century pagan cremation burial, and most of the items found in it bear traces of fire. The mound is located in the city of Chernigov (present-day Ukraine), it was excavated in 1872–1873, and a significant proportion of the finds was transferred in the beginning of the 20th century to the Historical Museum in Moscow (now the State Historical Museum). The object of study was a two-horned arrowhead with a shank. Some fragments of the object were lost, and traces of restoration work are present (Figure 5a). SR and neutron tomography of the arrowhead generally gave similar results; however, the contrast ratios of different image details differed (Figure 5b,c). The tomography showed the presence of a dense outer part in the object and a large number of voids of various shapes inside it. It can be concluded from SR images that the densest material is concentrated in a shell 0.3–0.5 mm thick. The voids occupy about 6%, while the shell makes up about 22% of the total volume of the object. According to neutron images, the shell material has a relatively low attenuation coefficient and, thus, contains less hydrogen. In contrast, the outer layer of an object on top of the shell and the material inside the dense shell attenuate neutrons more than the dense shell. The thermal neutron diffraction experiment showed that the material of the object consists of three phases: goethite α-FeO(OH), magnetite Fe_3_O_4_, and a small amount of hematite α-Fe_2_O_3_. According to the diffraction data, the object does not contain metallic iron. Aggregation of the data suggests that the shell material of the object is magnetite, which is most clearly visible in synchrotron images, while the outer and inner parts of the object are goethite and, possibly, amorphous products of iron corrosion.

From the observed picture of the structure of the object and the diffraction data, it can be concluded that a dense layer of mill scale formed on the surface of the arrowhead in the funeral pyre. At temperatures above 575 °C, which corresponds to the temperature of the flame of a large bonfire, iron is oxidized to wustite FeO, which is further oxidized upon cooling, forming magnetite, and, on the outer surface, there is a small amount of hematite [15]. It is the layer of magnetite and hematite that has protective properties. Formation of a layer of scale on the arrowhead ensured its preservation until the moment of excavation, since all the metal was destroyed by corrosion, which led, in particular, to the formation of an internal cavity. Due to such preservation of the arrowhead, it was possible to find and to reconstruct the original appearance of the ornament (Figure 5d,e) [14].

**Figure 6:**
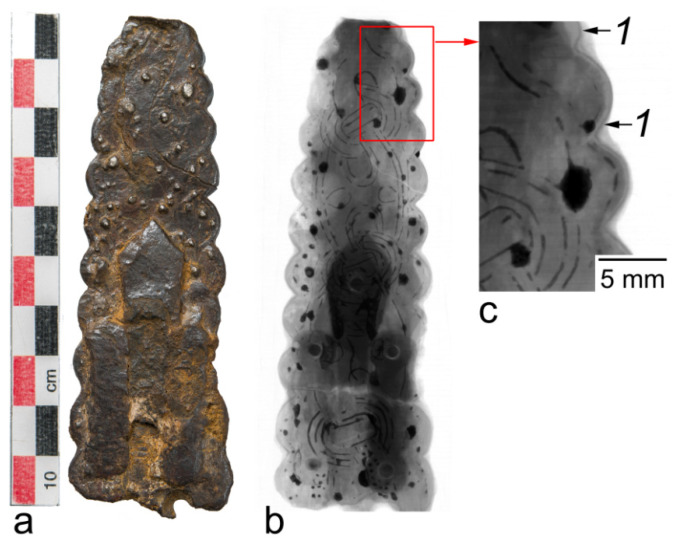
Study of the artifact from Chernaya Mogila: (**a**) photograph of the object; (**b**) SR radiograph; (**c**) enlarged part of the radiograph; 1—dense layer of mill scale.

**Figure 7:**
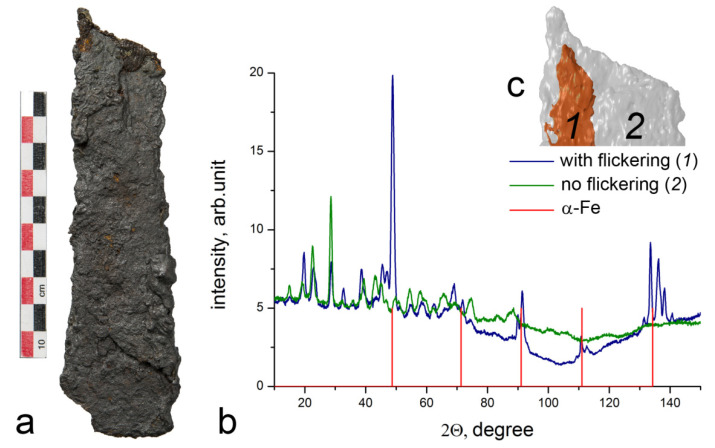
Detection of metallic iron in an artifact from Chernaya Mogila: (**a**) photograph of the object; (**b**) neutron diffraction pattern at two positions of the sample; the positions of the α-Fe lines are indicated; (**c**) reconstruction of the region of preservation of iron in the object.

Various aspects of the study of preservation state can be considered from the example of two other artifacts from the Chernaya Mogila burial mound [16]. The objects are oblong plates with dimensions 103 × 36 × 8 mm and 160 × 45 × 7 mm of unclear purpose. With the help of SR and neutron imaging, an ornament on them was revealed, which, together with data on other items of the collection, makes it possible to suggest that the studied artifacts are fragments of a scepter, a symbol of the power of its owner. According to the results of elemental analysis, the objects are almost entirely composed of iron corrosion products. SR imaging revealed a thin layer of mill scale near the surface of the objects, which could have contributed to the preservation of the objects similarly to the arrowhead (Figure 6). The artifacts also exhibit both transverse cracks 0.05–0.1 mm wide and delamination in the plane of the object, the width of which reaches 1 mm. Neutron tomography showed the presence of “flickering” regions due to the effect of diffraction of monochromatic neutrons on single crystals about 1 mm in size, which indicates that the metal of the objects is partially intact. Metal regions are located in the bulk of objects and have a longitudinal dimension of about 5 mm and a thickness of about 1–2 mm. Identification of one of these regions as a preserved metal was carried out using thermal neutron diffraction (Figure 7). A neutron beam of 0.167 nm wavelength, shaped by a 2(H) × 10(V) mm slit was directed to a section of the object in which the effect of neutron diffraction by single crystals was observed, as well as to a section where the effect was not observed. Accordingly, for the first section, diffraction lines of α-iron were recorded, and, for the second one, there were no diffraction lines of α-iron, while mainly magnetite and goethite were detected. No sample rotation was used; hence, the intensity ratio of reflections may be irregular.

## 4. Conclusions

The paper presented tomographic studies of metal artifacts of cultural heritage from the point of view of their preservation state. The examples reviewed demonstrate various preservation cases observed in research. Corrosion is a major process that threatens the integrity of metal archaeological objects. When examining corrosion damage, it is important to determine the depth of the corrosion layer, the propagation of corrosion deep into the metal, and, in cases of severe damage, the presence of the metal itself. Since corrosion products are mainly metal hydroxides, they contain hydrogen, which has a large scattering cross-section of thermal neutrons compared to other elements; thus, corrosion products are easily detected using neutron tomography. The impact of fire on the preservation of metal artifacts can be controversial. As a rule, fire destroys objects, leads to chemical transformation of a substance, and destroys the parts of an object made of fusible substances. On the other hand, fire can create a protective layer on the surface of an object that protects it from further destruction. Of great importance in this study was the observation of diffraction effects, which made it possible to reveal the presence of a coarse-grained metal structure. Essential information on the safety of objects can be extracted from the complementarity of neutron and X-ray or SR tomography data, supplemented by data on the elemental and phase composition of the material. Information on the preservation state of the artifacts is of great value for further decisions on their conservation and restoration [17].

## Data Availability

Not applicable.
